# Nematocidal Flavone-*C*-Glycosides against the Root-Knot Nematode (*Meloidogyne incognita*) from *Arisaema erubescens* Tubers

**DOI:** 10.3390/molecules16065079

**Published:** 2011-06-20

**Authors:** Shu Shan Du, Hai Ming Zhang, Chun Qi Bai, Cheng Fang Wang, Qi Zhi Liu, Zhi Long Liu, Yong Yan Wang, Zhi Wei Deng

**Affiliations:** 1 State Key Laboratory of Earth Surface Processes and Resource Ecology, Beijing Normal University, Beijing 100875, China; 2 Department of Entomology, China Agricultural University, Haidian District, Beijing 100094, China; 3 Analytical and Testing Center, Beijing Normal University, Beijing 100875, China

**Keywords:** nematicidal activity, *Arisaema erubescens*, *Meloidogyne incognita*, schaftoside, isoschaftoside

## Abstract

A screening of several Chinese medicinal herbs for nematicidal properties showed that *Arisaema erubescens* (Wall.) Schott tubers possessed significant nematicidal activity against the root-knot nematode (*Meloidogyne incognita*). From the ethanol extract, two nematicidal flavone-*C*-glycosides were isolated by bioassay-guided fractionation. The compounds were identified as schaftoside and isoschaftoside on the basis of their phytochemical and spectral data. Schaftoside and isoschaftoside possessed strong nematicidal activity against *M. incognita* (LC_50_ = 114.66 μg/mL and 323.09 μg/mL, respectively) while the crude extract of *A. erubescens* exhibited nematicidal activity against the root-knot nematode with a LC_50_ value of 258.11 μg/mL.

## 1. Introduction

Plant-parasitic nematodes are responsible for substantial economic losses to agricultural crops. Nematode management is generally based upon chemical treatments (soil fumigation), but environmental concerns and governmental regulations [1] are now resulting in a strong interest in nematicides of natural origin [2,3]. One alternative is to screen naturally occurring plant secondary compounds for appropriate activity. Many plant constituents and metabolites have been investigated for activity against plant-parasitic nematodes [4,5,6,7,8,9,10]. Varied nematicidal substances of plant origin such as triglycerides, sesquiterpenes, alkaloids, steroids, diterpenes and flavonoids have been identified in this way [2]. During our screening program for new agrochemicals from local wild plants and Chinese medicinal herbs, an ethanol extract of Chinese *Arisaema erubescens* (Wall.) Schott (Chinese cobra lily, Family: Araceae) tubers was found to possess strong nematicidal activity against the root-knot nematode, *Meloidogyne incognita* (Kofoid and White) Chitwood. *Meloidogyne incognita* is the most economically important and widely distributed nematode throughout China and a cause of considerable crop losses.

*A. erubescens* is widely distributed in China [11] and its tubers are used as a Traditional Chinese Medicine. The actions of the medicinal herb are to remove damp-phlegm, to dispel wind and arrest convulsions, and to promote the subsidence of induration and swelling [12]. Its pharmacological anticonvulsant and anti-cancer effects have been reproduced in a modern pharmacological study [13]. Due to the fact that it is a common Chinese herb used in medicine, the chemical constituents and bioactivities of *A. erubescens* tubers have been extensively studied and the known chemical constituents of this medicinal herb include calcium oxalate, paeonol, aurantiamide acetate, monoterpenoids, fatty acids, flavonoids, and alkaloids [14,15,16,17,18,19,20]. Ethanol extracts of * A. erubescens* tubers were found to possess insecticidal activity against the house flies (*Musca domestica*) [20]. The *n*-butanol extracts of *A. erubescens* tuber exhibited a quick knockdown molluscicidal activity against *Oncomlania hupensis* [21,22]. However, no bioactive compounds against nematodes have been isolated and identified from this Chinese medicinal herb. In this paper, we report the isolation and characterization of two natural compounds flavone-*C*-glycosides obtained from *A. erubescens* tubers by bioassay-guided fractionation and their nematocidal assessment against *M. incognita*.

## 2. Results and Discussion

### 2.1. Isolated Bioactive Compounds

**Figure 1:**
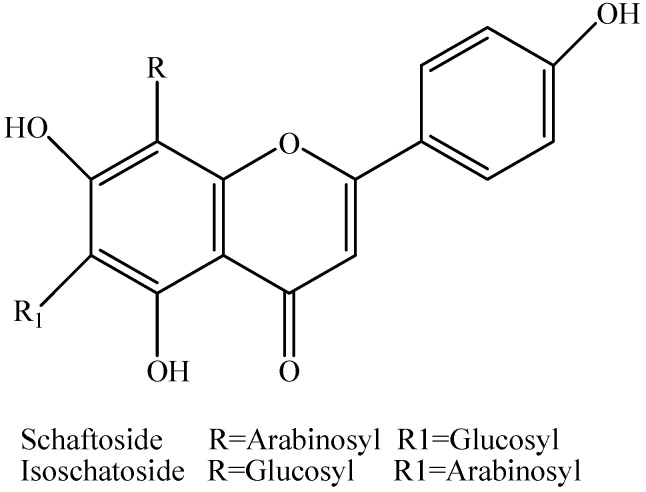
Structures of nematicidal flavone-*C*-glycosides isolated from *Arisaema erubescens* tubers.

Two bioactive compounds were isolated and based on bioassay-guided fractionation and identified based on their spectroscopic data and comparison with literature vales. Their chemical structures are given in Figure 1.

### 2.2. Nematocidal Activity

The ethanol extract of *A. erubescens* tubers exhibited toxicity against the root-knot nematode with a LC_50_ value of 258.11 μg/mL (Table 1). Compared with the positive control, carbofuran (LC_50_ = 72.29 μg/mL), the crude extract of *A. erubescens* tubers was 3.5 times less active against the root-knot nematode. However, considering carbofuran is a synthetic pesticide, this nematocidal activity of the crude extract of *A. erubescens* tubers is quite promising. Of the two identified active substances, schaftoside (LC_50_ = 114.66 μg/mL) was more toxic than isoschafoside (LC_50_ = 323.09 μg/mL) against the root-knot nematodes (Table 1). Schaftoside was two times more toxic against *M. incognita* compared with the crude extract and exhibits the same level of toxicity as the positive control carbofuran (Table 1). A literature survey shows that only one flavone-*C*-glycoside, lantanoside, exhibited toxicity against *M. incognita* and 90% mortality (24 h) was observed at a concentration of 1% (10 mg/mL approximately) [29]. Flavonoid glycosides have been reported to stimulate the probing of rice plant hoppers [30,31,32], whereas apigenin-*C*-glycosides such as schaftoside deterred feeding of *Nilaparvata lugen* [33]. Moreover, isoschaftoside from *Desmodium uncinatum* root exudates was found to inhibit growth of germinated *Striga hermonthica* radicles [34].

**Table 1:** Nematicidal activity of ethanol extract of *Arisaema erubescens* and isolated flavone-C-glycosides against *Meloidogyne incognita*.

Treatments	Concentrations (μg/mL)	LC_50_ (μg/mL)	95% Fiducial limits	Chi-Square Tests (χ^2^)
Ethanol extract	20.0-600.0	258.11	189.42-379.64	6.45
Schaftoside	20.0-600.0	114.66	52.27-160.64	6.98
Isoschaftoside	20.0-600.0	323.09	176.45-517.66	4.74
Carbofuran	25.0-400.0	72.29	37.86-117.97	13.57

## 3. Experimental

### 3.1. General

Melting points were measured on a Buchi 535 melting point apparatus and are uncorrected. FTIR spectra were recorded on a Magna IR^TM^ spectrometer 750. ^1^H- and ^13^C-NMR spectra were recorded on a JEOL JNM-AL300 spectrometer (300 MHz) using DMSO-d_6_ as solvent with TMS as internal standard. FAB-MS was determined on an APEX II (Bruker Daltonic Inc) spectrometer and UV was carried out on a Shimadzu UV-2450 spectrometer.

### 3.2. Plant Material, Extraction and Isolation of Active Ingredients

Dried tubers (4 kg) of *A. erubescens* were purchased from the Anguo Chinese Medicinal Herbs Market (Anguo 071200, Hebei Province, China). The species was identified, and the voucher specimens (BNU-CMH-Dushuahan-2009-08-25-006) were deposited at the Herbarium (BNU) of College of Life Sciences, Beijing Normal University. The tubers were ground to a powder using a grinding mill (Retsch Muhle, Germany), and the powder was extracted with 80% ethanol (50 L) at room temperature over a period of three weeks. The extract was evaporated to 1,200 mL under reduced pressure using a vacuum rotary evaporator, water (800 mL) was added and the extract was partitioned between ethanol-water and petroleum ether (3 × 2,000 mL). The petroleum ether extracts were evaporated to give a residue (8.0 g). The aqueous layer was re-partitioned with ether (3 × 2,000 mL) to provide a residue (8.0 g) after evaporation of ether. The aqueous layer was applied to a polyamide column (100-200 mesh, Jiangsu Changfeng Chemical Co., Ltd.), eluting with H_2_O and ethanol. The water-eluting fraction was further applied to a Macroreticular absorbing resin AB*-*8 (Tianjin Nanda Resin Technology Co., Ltd.), eluting with 10% ethanol, 30% ethanol and 50% ethanol to obtain fractions D_1_ (7.1 g), D_2_ (13.4 g) and D_3_ (1.3 g), respectively. D_2_ was further fractioned by polyamide column chromatography, Sephadex LH-20 (Pharmacia, Sweden), Toyopearl HW-40F (TOSOH (Shanghai) CO., Ltd.) and HPLC (Waters Delta Prep 4000) to obtain two bioactive compounds which was determined to be schaftoside (2.7 g) and isoschaftoside (51.0 mg). The structures of the compounds were elucidated based on mass spectrometry and nuclear magnetic resonance. 

### 3.3. Compound Characterization

*Schaftoside*. Amorphous yellow powder (EtOH), m.p. 222-224 °C. UV (λ^MeOH^ nm): 270, 330; IR (ν^KBr^ cm^−1^): 3423, 2926, 1649, 1576, 1356, 1217, 1053; FAB-MS (m/z): 565 (M^+^ + 1), 509 (M^+^-H^+^-3 × 18), 467 (M^+^-H^+^-2 × 18-60), 429 (M^+^-H^+^-134), 345 (M^+^-2H^+^-2 × 18-60-121), 307, 257, 219. ^1^H-NMR δ ppm:13.79 (s, 1H, 5-OH), 7.97 (d, *J* = 6.0 Hz, 2H), 6.92 (d, *J* = 6.0 Hz, 2H), 6.73 (s, 1H), 4.96 (d, *J* = 9.3 Hz, 1H, arabinose anomeric-H), 4.53 (d, *J* = 8.7 Hz, 1H, glucose anomeric-H), 4.00-3.00 (m, sugar-H). ^13^C-NMR δ ppm: 182.3 (C-4), 163.6 (C-2), 161.2 (C-7), 161.2 (C-5), 160.8 (C-4’), 153.4 (C-9), 128.8 (C-2’, 6’), 121.3 (C-1’), 116.0 (3’, 5’), 109.3 (C-6), 103.7 (C-8), 103.0 (C-10), 102.5 (C-3), 79.4 (G-3, 5), 74.9 (A-1), 74.3 (G-1), 73.7 (A-3), 70.3 (G-4, A-5), 70.0 (A-4), 69.8 (A-2), 68.4 (G-2), 60.8 (G-6). The MS, ^1^H- and ^13^C-NMR data were in agreement with the reported data [24,25,26].

*Isoschaftoside*. Yellowish powder (EtOH), m.p. 239-240 °C. UV (λ^MeOH^ nm): 270, 330. FAB-MS (m/z): 565 (M^+^ + 1), 547 (M^+^ + 1-18), 511 (M^+^ + 1-3 × 18), 475 (M^+^ + 1-90), 427 (M^+^ + 1-18-120). ^1^H-NMR δ ppm:13.82 (1H, s, 5-OH), 10.29 (1H, s, 7-OH), 9.19 (H, s, 4’-OH), 8.32 (2H, d, *J* = 7.9 Hz, 2’, 6’-H), 6.92 (2H, d, *J* = 7.9 Hz, 3’, 5’-H), 6.87 (1H, s, 3-H), 4.77 (anomeric-H), 4.72 (anomeric-H), 4.70-3.00 (m, sugar-H). ^13^C-NMR δ ppm: 183.2 (C-4), 165.2 (C-2), 162.1 (C-7), 161.9 (C-4), 159.1 (C-4’), 156.1 (C-9), 130.6 (C-2’, 6’), 121.9 (C-1’), 116.8 (C-3’, 5’), 108.9 (C-6), 106.0 (C-8), 104.7 (C-10), 102.9 (C-3), 81.5 (G-5), 76.6 (G-3), 75.1 (A-1), 74.7 (G-1, A-3), 71.0 (A-5), 70.5 (G-4), 70.1 (A-4), 69.3 (A-2), 69.1 (G-2), 62.2 (G-6). The MS, ^1^H- and ^13^C-NMR data were in agreement with the reported data [24,25,26,27,28].

### 3.4. Nematicidal Assay

Second stage juveniles (J2) of *M. incognita* were obtained from a pure culture that was previously initiated by egg masses and propagated on tomato (*Solanum lycopersicum*) in the glasshouse. Egg masses were hand picked using sterilized forceps from heavily infected roots (40 days after incubation). These egg masses were washed in distilled water, placed in 15 mesh sieves (8 cm in diameter) containing crossed layers of tissue paper in Petri-dishes with water just deep enough to contact the egg masses and incubated 25-26 °C to obtain freshly hatched second stage juveniles (J2). Only juveniles collected within 48 h were used. Range-finding studies were run to determine the appropriate testing concentrations. A serial dilution of ethanol extract of *A. erubescens* (five concentrations, dissolved first in 10 μL ethanol) and pure compounds (five concentrations) was prepared in H_2_O solution with 2% DMSO. Aliquots of H_2_O (20 µL) containing ca. 30 juveniles (J2) were transferred to vials to which 980 µL of the solution containing ethanol extract or pure compounds was added. The vials were kept on a hood at 25 °C. The inactive nematodes were counted every 24 h for 72 h. After the last count, the inactive juveniles were maintained in distilled H_2_O for 24 h to observe their revival. Six repetitions for each treatment were performed using H_2_O and a 2% DMSO in H_2_O solution as well as a 2% DMSO in H_2_O solution containing 10 μL ethanol H_2_O as control. The experiments were repeated in three times. Results from all replicates for the pure compounds and ethanol extract were subjected to probit analysis using the PriProbit Program V1.6.3 to determine LC_50_ (median lethal concentration) values and their 95% confidence intervals (CI 95%) [23].

**Figure 2:**
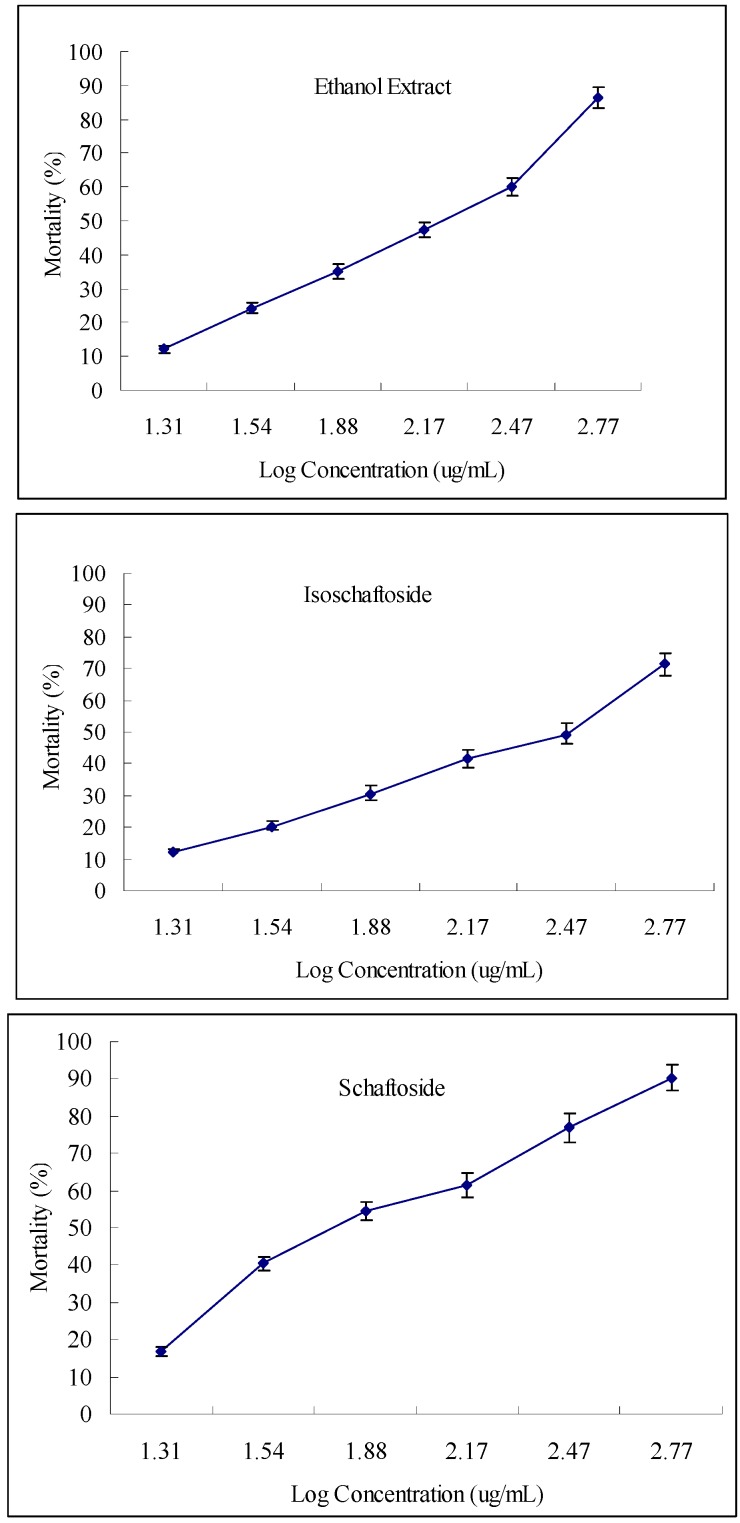
Dose-response curves of *Meloidogyne incognita* (J2) treated with ethanol extract of *A. erubescens* and the two flavone-C-glycosides.

## 4. Conclusions

Based on mass screening of medicinal herbs, the ethanol extract of *A. erubescens* tubers was found to possess strong toxicity against the root-knot nematode species *M. incognita*. Two nematicidal flavone-*C*-glycosides were isolated and identified from the extract by bioactivity-guided fractionation. Schaftoside exhibits the same level of toxicity against *M. incognita* as the positive control (the commercial pesticide carbofuran). Moreover, the compound was two times more toxic against *M. incognita* than the crude extract. These findings suggest that the ethanol extract of *A. erubescens* tubers and two isolated compound show potential for development as natural pesticides for the control of nematodes.
